# Nanoenergetic Materials: Preparation, Properties, and Applications

**DOI:** 10.3390/nano10122347

**Published:** 2020-11-26

**Authors:** Djalal Trache, Luigi T. DeLuca

**Affiliations:** 1 Teaching and Research Unit of Energetic Processes, Energetic Materials Laboratory, Ecole Militaire Polytechnique, BP 17, Bordj El-Bahri, Algiers 16046, Algeria; 2 Department of Aerospace Science and Technology, Politecnico di Milano, 20, 156 Milan, Italy; luigi.t.deluca@gmail.com

Energetic materials (EMs) are considered to be pure components or mixtures of chemical substances, which consist of both fuel and oxidizer that could release a large amount of energy or gas upon ignition. EMs can broadly be classified into propellants, explosives, and pyrotechnics with a wide range of applications in ordnance, rockets, missiles, space technology, fireworks, gas generators, automobile airbags, deconstruction, welding, and mining, to cite a few [1]. Explosives generate supersonic detonation velocity but low energy density, whereas propellants and pyrotechnics provide high energy densities by subsonic deflagration process. Typically, EMs can be produced as either monomolecular materials or as composites. The first class, which contains reactants within the same molecule, exhibits a fast reaction process but presents low performance, whereas the second class, which displays better performance, suffers from the slow reaction process mainly due to the limited mass transport rate between species. Several additives such as catalysts, coolants, stabilizers, and plasticizers, in few percent ratios, could be added to the EM formulations to improve their peculiar features and tailor their performance [2].

The advancement in the synthesis approaches and the advent of material characterization tools at multiple length scales have pushed the energetic materials community to explore new opportunities. During the past two decades, several significant achievements in research on nanoenergetic materials (nEMs) have been realized, thanks to the technological novelties in the field of nanoscience and nanotechnology. The principle of nanoenergetics is the enhancement of the specific surface area and intimacy with chemical components to improve the reaction rate while reducing the ignition delay at an acceptable level of safety. Nanoenergetics started with the manufacturing of nano-sized metal particles, mainly aluminum, which was mainly used for rocket propulsion, since the second half of the 20th century. During the last two decades, the physical mixing of oxidizers and fuels is considered as the second stage of the development of nanoenergetics at the nanoscale for which the diffusion distances between the chemical species is improved and the surface-over-volume ratio is enhanced, currently reaching the advanced third stage, where modern technologies, which allowed producing novel types of reactive nanocomposites structures and morphology with tunable features, are applied [3,4].

nEMs, which are composed of nano-sized fuel and oxidizer with or without additives, have been found to be potential sources of extremely high heat release rates and tailored burning rates, reliability, and extraordinary combustion efficiency. Nowadays, they play a vital role in widespread applications such as miniaturized electro-explosive devices, the attitude control of micro/nano satellites, and actuation in lab-on-a-chip devices, to name a few. The improvement of properties and the discovery of new functionalities and methodologies are key goals that cannot be reached without a better understanding of the preparation, characterization, manufacturing, and properties that constitute the starting points of the design of specific and adequate systems. The investigation of nanoenergetic materials has demonstrated both academic as well as technological importance and offered great research opportunities within cross-disciplinary areas.

In this framework, the present Special Issue in *Nanomaterials* aims to further contribute to the momentum of research and development in nanoenergetic materials, by featuring eleven (11) original research articles, two (2) review articles as well as one (1) short communication, authored and reviewed by experts in the field. This targets a broad readership of materials scientists, chemists, physicists, and nanotechnologists, among others. The potential topics address issues of the synthesis, characterization, properties, modifications, and applications of nanoenergetic materials as well as the incorporation of nano-additives (e.g., catalysts) for energetic material formulations. Most of the research papers highlight theoretical concepts and practical approaches of interest for real-world applications related to nanoenergetic materials.

Four interesting research papers dedicated to the synthesis, modification and characterization of nanoscale ingredients for new energetic formulations are published in this Special Issue. Luo et al. prepared an energetic composite fiber, in which 2,6-diamino-3,5-dinitropyrazine-1-oxide (LLM-105) nanoparticles are intimately incorporated with a nitrocellulose/glycidyl azide polymer (NC/GAP) fiber through the electrospinning method [5]. Compared to pure NC/GAP and LLM-105 nanoparticles, the obtained insensitive three-dimensional nanofibers with a large specific surface area exhibited a lower decomposition temperature but a higher decomposition rate. Such nanofibers displayed outstanding performance with a higher combustion chamber temperature and improved specific impulse as well. The authors claimed that such nanofibers might find potential application in the field of solid rocket propellant systems. One paper by Wang et al. explored the ultra-low temperature spray method to produce 2D network structures of nanoscale ammonium perchlorate (AP) and ammonium nitrate (AN) [6], which are widely used as oxidizer of solid rocket propellants [7]. The authors demonstrated that the obtained nano-AP was more sensitive than the raw micro-AP, whereas either nano- or micro-sized particles of AN were both insensitive to friction and impact stimuli. They reported that the thermal decomposition of nanometric AP and AN occurred at lower temperatures with respect to their raw materials, respectively. The decomposition products of nano-AP corresponded to NO_2_, N_2_O, HCl, H_2_O with a small amount if NOCl, whereas those of nano-AN were mainly N_2_O and H_2_O with a small amount of NH_3_. The study by Dobrynin et al. assessed the potential of supercritical antisolvent (SAS) processing to produce nano-sized nitrocellulose (NC) [8], which is a workhorse of numerous energetic formulations [9]. The authors prepared nano-NC with an average particles size of 190 nm from raw NC of 20 μm in diameter. The obtained nano-NC displayed low friction sensitivity and lower decomposition temperature compared to the raw NC. The authors prepared nano-NC/carbon nanotubes/nano-Fe_2_O_3_ nanocomposites for which the combustion process exhibited an increase in the burning rate of 20% at 12 MPa compared to the pure nano-NC. In addition, the authors revealed that the employment of SAS processing to produce the nanocomposite offered higher performance, lower sensitivity and better stability compared to nanocomposites prepared by conventional dry mixing. In another research work, the Dreizin research group prepared fuel-rich composite powders combining elemental Si with metal fluoride oxidizers BiF_3_ and CoF_2,_ using the common approach of arrested reactive milling [10]. The reactivity assessment of the obtained powders was carried out by means of thermo-analytical techniques in both inert argon (Ar) and oxidizing (Ar/O_2_) atmospheres. In addition, the obtained powders were ignited by an electrically heated filament using CO_2_ laser as an ignition source under ambient atmosphere. They mentioned that both composites lead to fast ignition and effective oxidation under oxidizing atmosphere, while elemental Si could not ignite using either laser or heated filament under the same conditions. It was also demonstrated that the combustion process, which occurred at lower temperature, is more effective on BiF_3_ for which both oxidation and fluorination occur nearly simultaneously. However, during the use of CoF_2_, it was found that the oxidation of Si occurs before its fluorination.

Four papers are dedicated to the evaluation of the reactivity and performance of some nanoenergetic materials through controllable manufacturing and/or modifications approaches. A paper by the Rossi research group introduced one area of interest by describing the technology of the deposition of Al/CuO multilayers through sputter-deposition [11]. Such multilayers are expected to be employed as tunable igniters and actuators for defense, space and infrastructure safety applications. The same research group described in another paper a joint experimental/theoretical investigation dealing with the aging of reactive Al/CuO nanolaminates through the assessment of the structural changes as well as the combustion features [12]. The group reported that the nanolaminates remained stable even after decades of storage at ambient temperature. The authors revealed that the aging transition occurred at 200 °C for 14 days for which the interfacial modification is attributed to the stack dimensional characteristics. They also demonstrated that the burn rate of Al/CuO nanolaminates with bilayer thickness greater than 500 nm was not affected, whereas it decreased by ~25% for a thickness of 300 nm. In another study, Guo et al. designed prominent nano-Al/MoO_3_ metastable intermolecular composite (MIC) chips with the homogeneous distribution of particles through a suitable and high-effective electrophoretic deposition (EPD) method at room temperature and under ambient pressure conditions [13]. The obtained nano-MIC chips exhibited interesting heat-release performance. It was shown that the MIC ship initiator could be successfully ignited with a typical capacitor charge/discharge ignition device, displaying excellent detonation performance. The authors claim that the developed fabrication method is fully compatible with micro-electromechanical systems, which can be employed for micro-ignition/propulsion applications. The report by Vorozhtsov et al. proposed an interesting approach, which is based on the coating process of nano-aluminum using hydroxy terminated polybutadiene (HTPB), to enhance the performance of the nano-fuel within a propellant formulation [14]. The authors demonstrated that the coating process, which maintains a high reactivity of nano-Al, has been successfully performed. The authors report that the prepared composite solid propellant based on nano-Al coated with HTPB provided a higher burning rate with an increase in the burning stability at low pressure compared to the propellant supplemented with nano-Al without HTPB coating.

Another increasing area of interest in nanoenergetic materials addressed in this issue concerns the incorporation of various additives such as catalysts to tailor the properties and improve the performance of energetic formulations. A paper by the Yan research group reported on the analysis of the gaseous products during the decompositi0on of ammonium perchlorate (AP) supplemented with graphene oxide (GO)-based additives through the employment of a tandem analytic tool, which consists of thermogravimetry coupled with mass spectrometry [15]. They proved that the GO-based catalysts improved the catalytic decomposition efficiency due to the capability in increasing the conversion rate of NH_3_ and H_2_O through the excessive O elements being transferred to react with NH_4_^+^, which enhanced the decomposition heat. The same research group investigated the morphology, particle size, and composition of condensed combustion products (CCP) during the combustion of modified double-base solid propellants, which contain nano-catalysts, under various pressures [16]. The authors report that the average particle size of CCPs with various morphologies decreased with the increase in pressure. The surface of CCP particles contained various chemical elements such as C, N, Al, Cu, Pb and Si. In another research work, Yao et al. prepared bamboo leaf-like CuO and flaky-shaped CuO by the hydrothermal method, which are combined with Al nanoparticles through ultrasonic dispersion methods [17]. The obtained composites have been assessed as catalysts of nitrocellulose. It was demonstrated that the decomposition process of NC with or without catalysts follows the same kinetic mechanism as the Avrami–Erofeev equation. The authors proved that the microstructure of CuO affected the thermolysis process, where the presence of flaky-shaped CuO/Al lead to an easier ignition of NC. Within the same subject, the Trache research group produced hematite nanoparticles decorated on carbon mesospheres and assessed their effect on the thermal decomposition of nitrocellulose [18]. It was revealed that the obtained nanoparticles had a minor effect on the decomposition temperature of nitrocellulose, whereas an obvious decrease in the activation energy was acquired. Moreover, the decomposition reaction mechanism of NC is influenced by the incorporation of the nano-catalyst.

Metal nanoparticles have receive tremendous attention from the scientific community [19,20]. They display interesting features in various applications in the combustion and explosion processes of energetic materials. For such a type of nanomaterials, two interesting review papers have been published in the current issue. The first review article by Pang et al. describes the recent advance in the preparation, characterization and performance of Al-based nano-sized composite energetic materials (CEMs) [21]. The authors emphasize the most important approaches that are currently applied worldwide to improve the performance of Al-based nano CEMs, which are revealed to differ from those based on micro CEMs. The remaining issues and challenges for the future research directions have been deeply discussed. The last paper in the issue by Zarko and Glazunov is dedicated to an overview of experimental methods used to measure the ignition and combustion characteristics of metal nanoparticles [22]. The authors focus on the methods employed to determine the nanoparticle size, their heat-exchange parameters as well as the ignition delay and combustion time. They reveal that despite the advance of the analytic method to investigate the metal nanoparticles features, other issues exist concerning the comparison of the data of ignition and burning time with respect to particle size dependencies. They recommend continuing the development of effective approaches in the future to correctly describe the particles’ dynamic behavior.

In summary, the present Special Issue advances not only our understanding of the emerging and significant role of nanoenergetic materials for the future of pyrotechnical science, but also that of challenges and the future research directions to fully explore their potential features according to their practical utilization. Despite the fact that we are still struggling to translate the fundamental advances reported in the scientific literature into tangible applications, it is expected that this Special Issue will encourage multidisciplinary research activities on nanoenergetic materials to overcome the current technological limitations. Thus, further experimental, simulation and calculation works should be carried out in the future through the focus on the scaling-up of the systems, the economic viability, stability and aging, coating and dispersion, material safety, hazard level during manufacturing, recycling needs, and the control of ignition processes. Finally, it is anticipated that nanoenergetic materials as the next generation of materials will be fruitfully integrated into defense, special and other civilian fields.
